# Retained foreign body misdiagnosed as a low flow vascular malformation: A case report

**DOI:** 10.1016/j.radcr.2023.10.007

**Published:** 2023-10-30

**Authors:** Daniel Armstrong, Robert Owens, Terri Carlson, Brian Ching

**Affiliations:** a Uniformed Services University of the Health Sciences, 4301 Jones Bridge Rd, Bethesda, MD, 20814, USA; b Department of Radiology, Tripler Army Medical Center, 1 Jarrett White Rd, Honolulu, HI, 96859, USA; c Department of Plastic and Reconstructive Surgery, Tripler Army Medical Center, 1 Jarrett White Rd, Honolulu, HI, 96859, USA

**Keywords:** Arteriovenous, Foreign body, Malformation, Splinter, Trauma, Venous

## Abstract

A 21-year-old woman presented with 1 year history of progressive dorsal right foot pain with no recollection of trauma. The physical exam did not reveal any unusual appearance to the plantar or dorsal skin surfaces. Specifically, no scars were seen. Foot radiographs were unremarkable. The presumed etiology after Doppler ultrasound (US) and MRI was a likely venous or venolymphatic malformation. She received 2 rounds of sclerotherapy 12 months apart with transient symptomatic clinical improvement. After the second sclerotherapy treatment, repeat MRI revealed dorsal extension of the lesion with skin involvement, prompting referral for surgical intervention. At surgery, a 3 cm wood splinter was found surrounded by granulation tissue. After showing the patient the resected splinter, she recalled stepping on a wooden broomstick that punctured the bottom of her foot in her childhood. She stated she also remembered pulling a splinter out of her foot. This case demonstrates the unusual and rare appearance of a chronic retained foreign body creating a cystic lesion in the foot presumed to be a low-flow vascular malformation on US and MRI.

## Introduction

Venous malformations are a rare form of congenital vascular malformation present in approximately 1-2 per 10,000 births [1]. These malformations typically present in childhood or adolescence as a blue compressible mass when they are located superficially, and later when intramuscular. They often cause pain and functional impairment depending on their location, degree of thrombosis, and mass effect. Additionally, they can present a cosmetic concern. Initial diagnostic imaging typically includes duplex ultrasound, with about 20% of cases exhibiting calcifications (phleboliths) [2]. Doppler flow is detected in most cases but may be absent. MRI is typically performed after ultrasound using T1 spin-echo and T2 fat-saturation sequences, along with postgadolinium sequences when necessary [2]. T2 hyperintensity is a typical finding. Angiography is also performed when sclerotherapy is used. Conservative measures are often used for clinically benign lesions, while sclerotherapy is the preferred treatment for painful or functionally limiting lesions [3]. Multiple treatments may be necessary to achieve resolution. While trauma is occasionally associated with the development of high-flow arteriovenous malformations, it is not typically associated with venous or lymphatic malformations [2]. Trauma-induced soft tissue masses are often found adjacent to prominent bones [4]. Characterization of cystic lesions in the extremities can be difficult, and MRI with contrast is indicated when a cystic-appearing lesion is identified in an atypical location or has atypical features [5]. Post-traumatic vascular malformations are uncommon, with spongiform soft-tissue lesions being the more common mimickers [6]. The clinical presentation and imaging appearance of vascular malformations are variable. Both clinical and imaging findings are extremely important in diagnosing vascular malformations and possible mimic lesions.

## Case

**Fig. 1:**
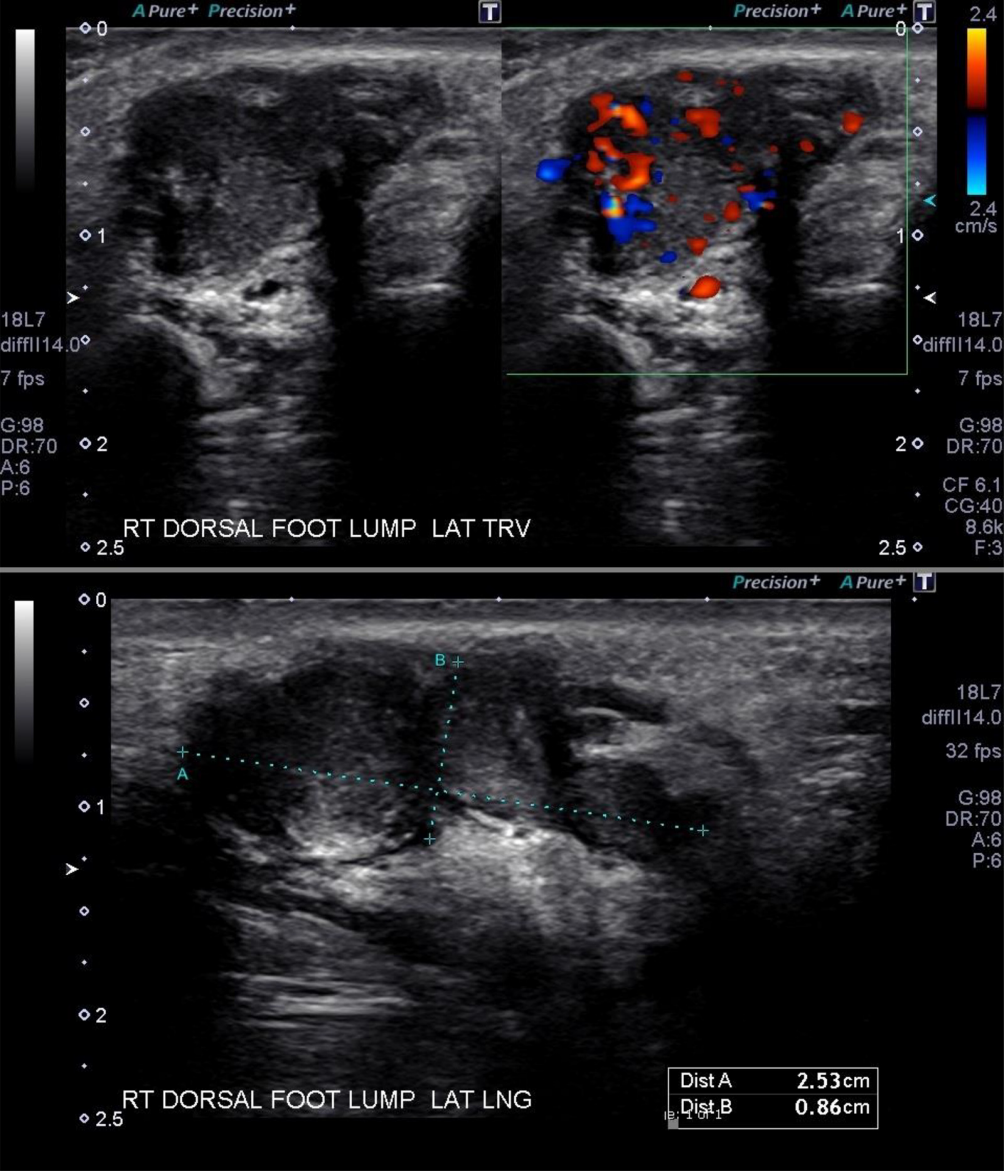
Lateral transverse without (upper left) and with (upper right) color Doppler ultrasound images demonstrate irregularly shaped cystic lesions with intralesional echogenic tissue with color Doppler. Longitudinal (lower) images demonstrate the longitudinal size of the lesion as seen above the level of the metatarsals.

**Fig. 2:**
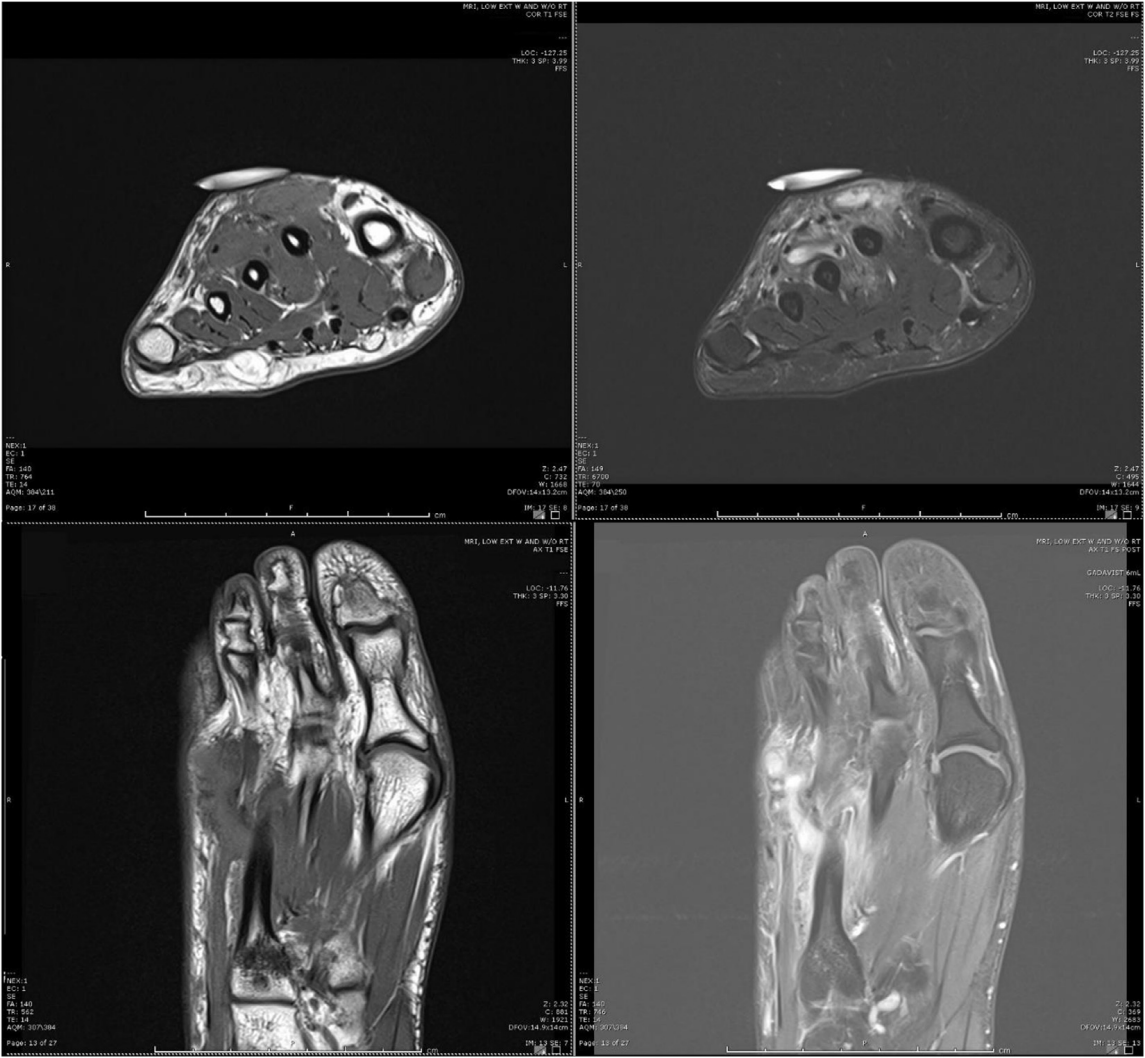
Coronal (above) and axial (below), pre (left), and post (right) gadolinium images demonstrate enhancing tissue and areas of fluid vs serpentine vessels between the second and third metatarsal shafts at the dorsal aspect.

A 21-year-old woman presented to her primary care physician with right foot pain, functional impairment, and a soft, mobile, nodular mass on the dorsum of her right foot. The US revealed 2 irregularly shaped cystic lesions with intralesional echogenic soft tissue with an arterial and venous flow on Doppler (Fig. 1). For further evaluation, an MRI with contrast was performed. Multiplanar T1, fast spin-echo T2, fat-suppressed T1, and fat-suppressed T1 postgadolinium images were obtained with a 3 Tesla magnet. These images demonstrated a T2 hyperintense lobulated lesion with gadolinium enhancement and extension between the second and third metatarsal shafts (Fig. 2).

Interventional Radiology was then consulted as the US and MRI features best fit with a low-flow vascular malformation. The lesion morphology was very unusual, and the patient was specifically asked during the Interventional Radiology consultation if she had any history of trauma to her right foot in the past, which she denied. Physical exam was notable for an approximately 1 cm fluctuant nodular lesion of the dorsum of the foot between the second and third metatarsals that was mildly tender to palpation. There was no skin color change or scar about this lesion nor the plantar aspect of her foot opposite the dorsal nodule.

**Fig. 3:**
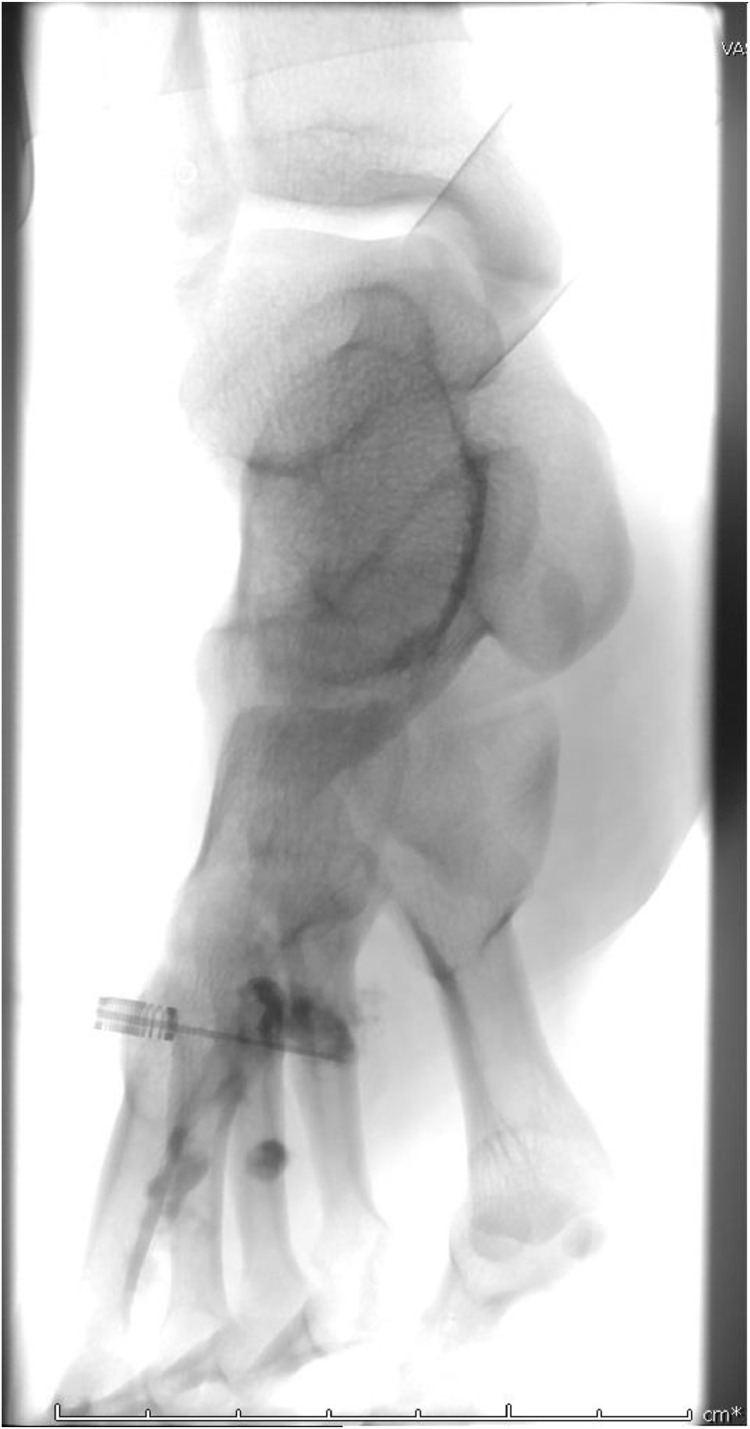
Percutaneous venogram demonstrating serpentine filling of the lesion with minimal appearing venous outflow.

**Fig. 4:**
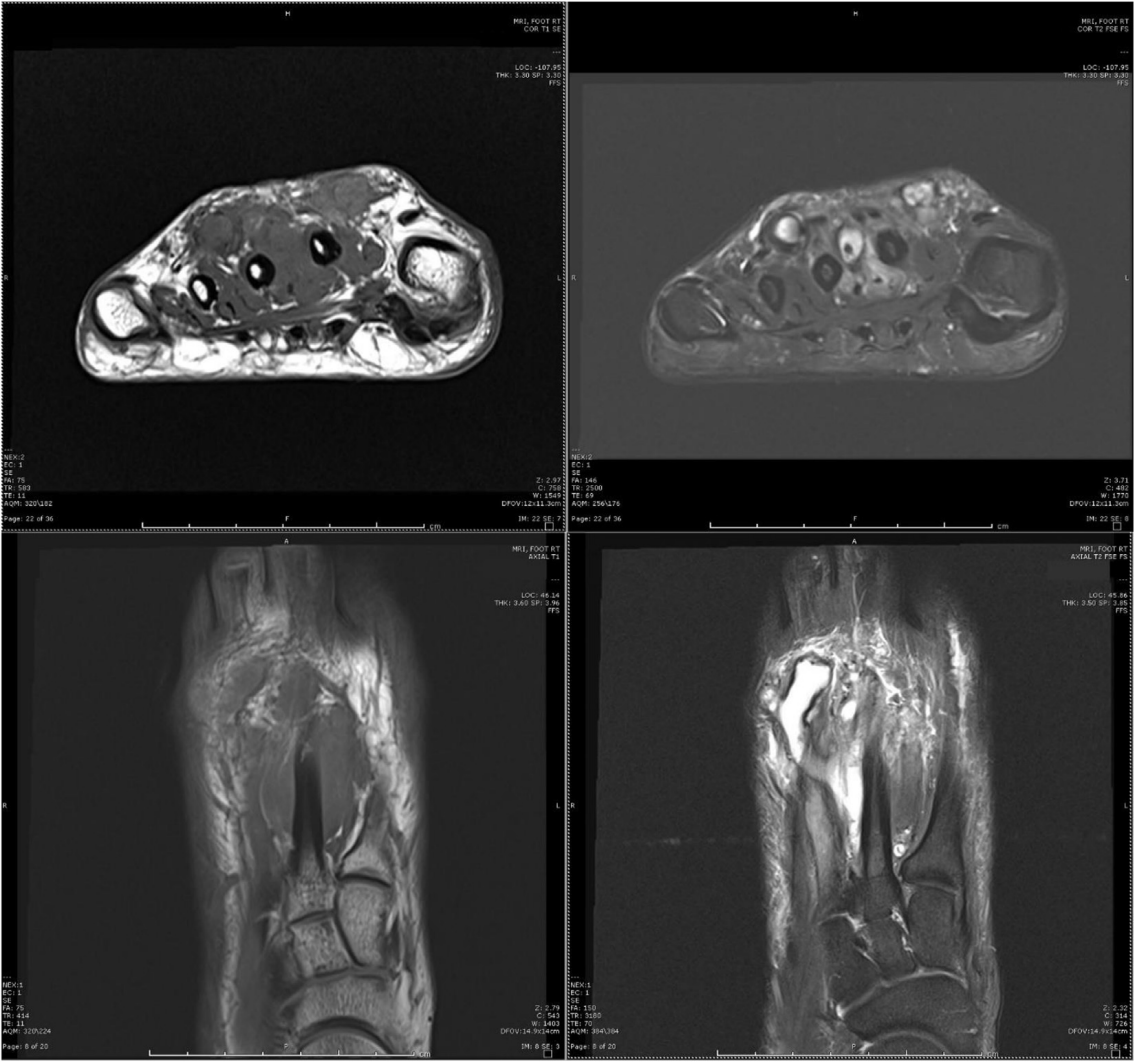
Coronal (above) and axial (below), pre (left), and post (right) gadolinium images 1 year after initial treatment redemonstrate enhancing soft tissues between the second and third metatarsal shafts at the dorsal aspect and more pronounced serpentine appearing presumed vessels.

The patient underwent sclerotherapy with 1.8 mL of doxycycline at 10 mg/mL. The associated venography demonstrated serpentine filling of the lesion with minimal venous outflow (Fig. 3). She reported moderate improvement in pain at subsequent follow-up visits. However, she did not achieve complete symptom resolution, and after several months she experienced a return of pain to pre-treatment levels. Additionally, she had an interval increase in size of the lesion on MRI (Fig. 4).

**Fig. 5:**
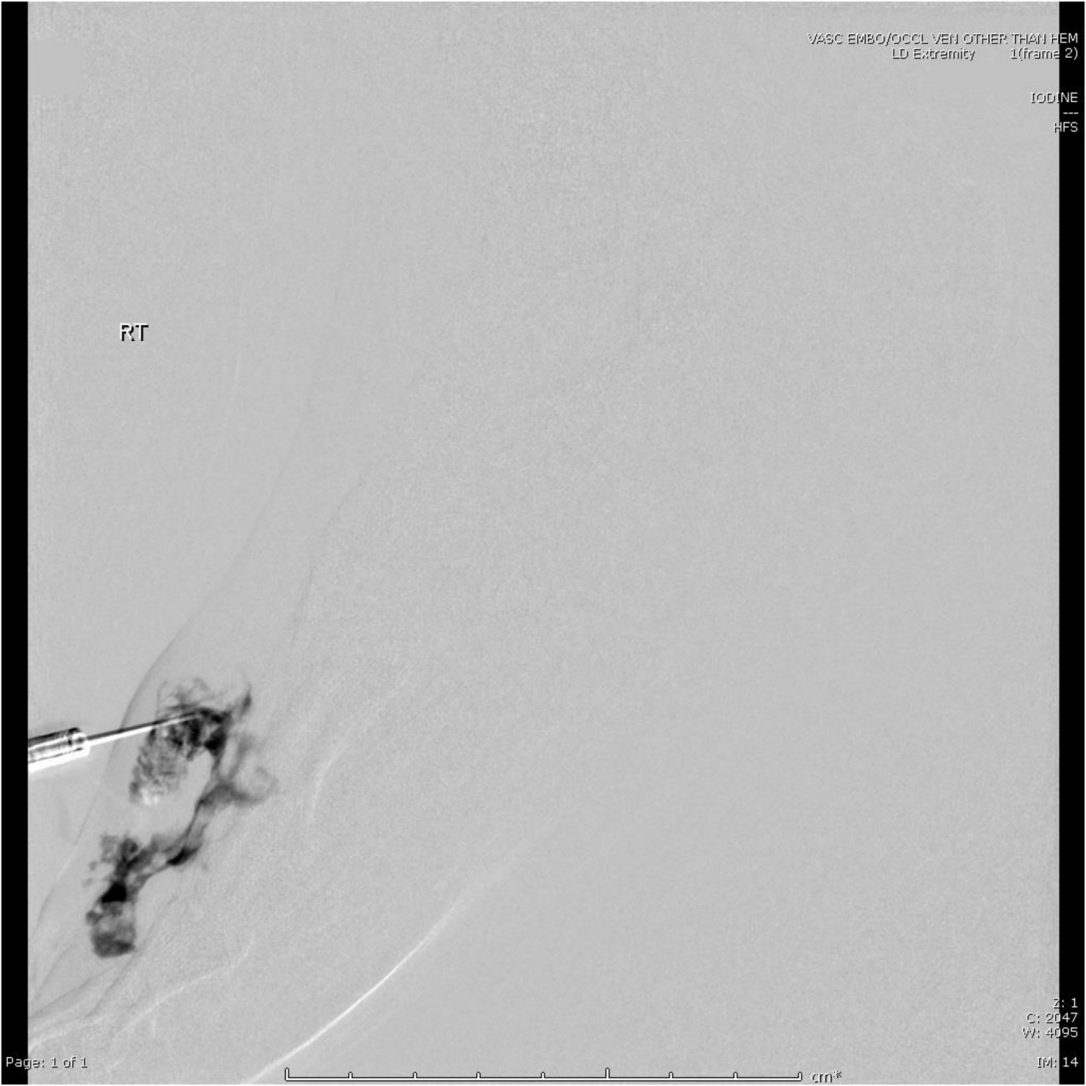
Digital subtraction angiography of the lesion at the second sclerotherapy treatment, redemonstrating the serpentine appearing vessels/channels and minimal appearing venous outflow.

**Fig. 6:**
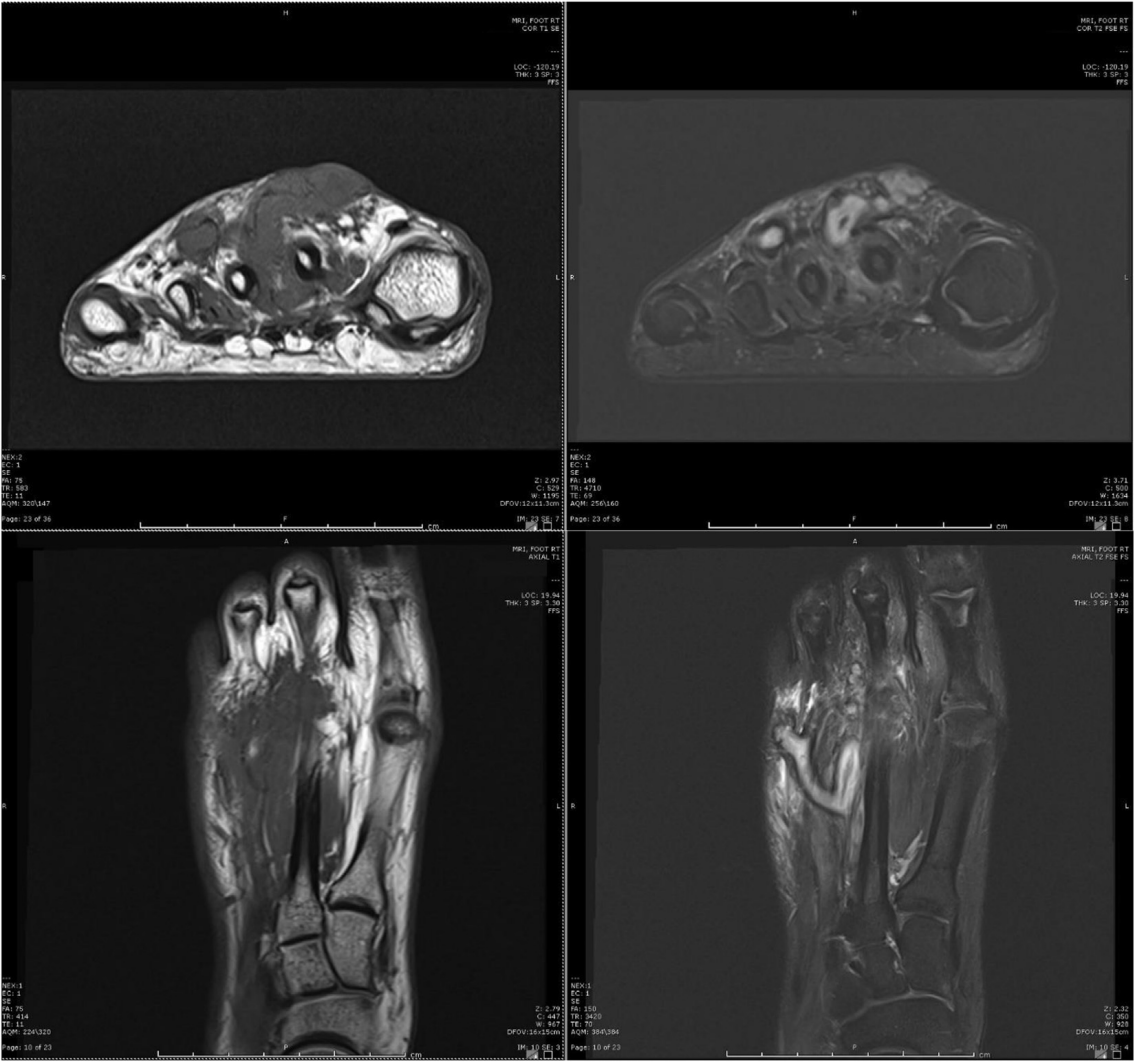
Coronal (above) and axial (below), pre (left) and post (right) gadolinium images 1 year after second sclerotherapy, and 2 years after the initial sclerotherapy redemonstrate enhancing soft tissue between the second and third metatarsal shafts with dorsal involvement. Persistent areas of fluid presumed to be vascular channels. Note the extension of the lesion over the second metatarsal shaft now approximates the skin.

**Fig. 7:**
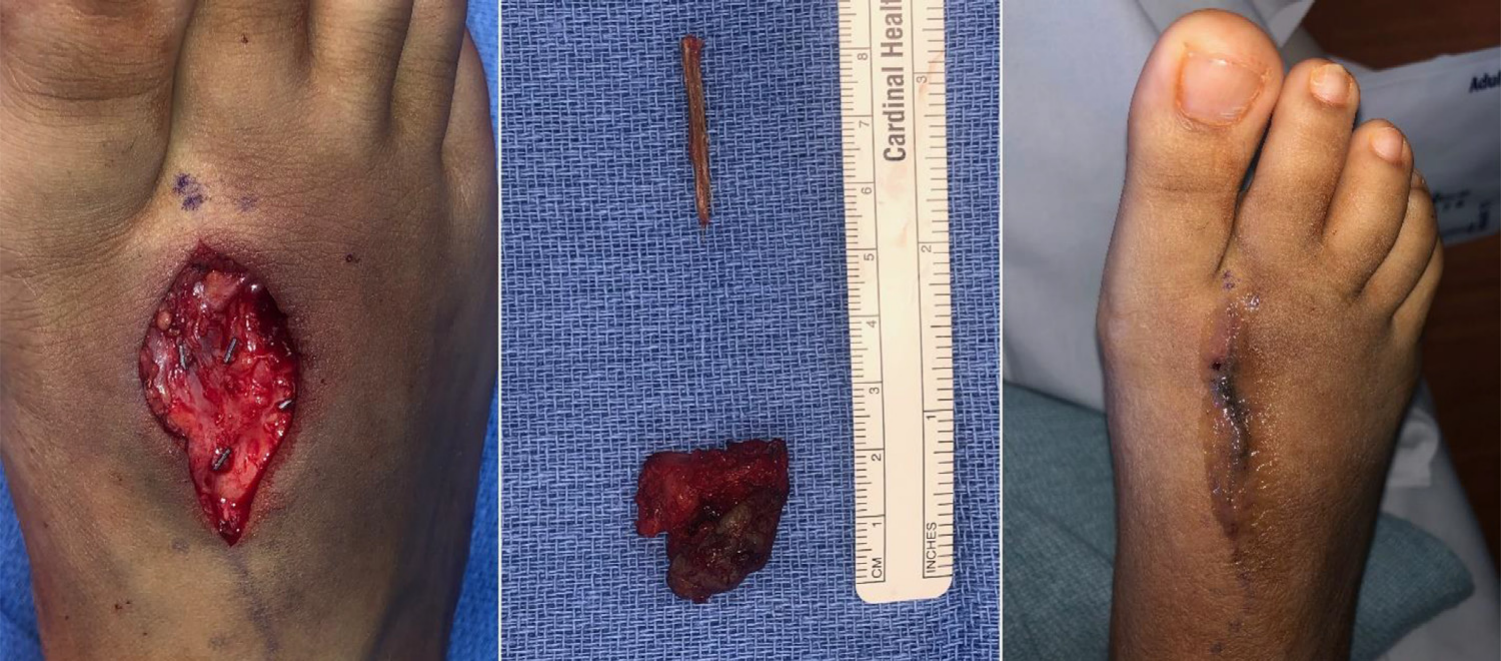
Excision of the lesion revealed a 3 cm wood splinter and mass of thrombosed vascular tissue (center).

A second sclerotherapy treatment was performed 1 year later with an additional 1.8 mL of doxycycline, with follow-up exams demonstrating a similar pattern of partial symptom resolution followed by recurrence (Fig. 5). Follow-up MRI showed expansion of the lesion dorsally with new involvement of the skin, correlating with a protuberant erythematous 1 cm lesion over the second metatarsal (Fig. 6). Plastic surgery was consulted, and she subsequently underwent excision of a 2.5 cm x 2.5 cm gelatinous, seropurulent mass surrounded by thrombosed vasculature between the second and third metatarsal shafts with plantar extension correlating to the MRI findings. Interestingly, a 3 cm wooden splinter was found within the mass (Fig. 7).

Additionally, after further detailed inspection of the plantar aspect of her foot in the operating room, a faint skin defect suspicious for a remote puncture site was identified. Discussion with the patient of the findings following surgery prompted her to recall an event from her early childhood in which she experienced intense pain after stepping on a broomstick but was unaware a portion of the wooden splinter that penetrated her foot was not completely retrieved.

## Discussion

Several aspects of this case complicated the correct diagnosis. The most critical information not revealed until surgery was a history of penetrating trauma to the foot at the site of her lesion. The length of time this foreign body was present in the foot without significant symptoms, the presentation on imaging, and the partial response to sclerotherapy also confounded the correct diagnosis.

The US and MRI with contrast were concordant but not classic in appearance for a low flow vascular malformation. While multiple rounds of sclerotherapy are frequently required to achieve symptom resolution, it was unusual that her symptoms were so poorly controlled after 2 treatments. This, coupled with the imaging findings of interval increase in size and dorsal migration led to surgical exploration. The wood splinter likely penetrated the plantar skin and migrated dorsally between the second and third metatarsals where it was clinically dormant for many years. The orientation and location of the splinter were likely parallel to the tendons, masking its appearance. The chronic granulomatous foreign body reaction and resultant neovascularization ultimately led to the clinical and imaging findings of this case.

This case demonstrates the importance of a comprehensive history and physical exam when dealing with an unusual appearing low flow lesion on US and MRI. Re-assessing the history and physical after no considerable improvement in symptoms from sclerotherapy treatments could have helped differentiate a chronic foreign body reaction mimicking a low flow vascular lesion.

## Patient consent

Written informed consent was obtained from the patient to publish this case report.
